# Oncogenic mechanisms of *HOXB13* missense mutations in prostate carcinogenesis

**DOI:** 10.18632/oncoscience.322

**Published:** 2016-10-31

**Authors:** Marta Cardoso, Sofia Maia, Paula Paulo, Manuel R. Teixeira

**Affiliations:** ^1^ Cancer Genetics Group, IPO Porto Research Center (CI-IPOP), Portuguese Oncology Institute of Porto (IPO Porto), 4200-072 Porto, Portugal; ^2^ Department of Genetics, Portuguese Oncology Institute of Porto (IPO Porto), 4200-072 Porto, Portugal; ^3^ Institute of Biomedical Sciences Abel Salazar (ICBAS), University of Porto, 4050-313 Porto, Portugal

**Keywords:** HOXB13, germline mutations, prostate cancer, *in vitro* assays

## Abstract

The recurrent germline mutation *HOXB13* p.(Gly84Glu) (G84E) has recently been identified as a risk factor for prostate cancer. In a recent study, we have performed full sequencing of the *HOXB13* gene in 462 Portuguese prostate cancer patients with early-onset and/or familial/hereditary disease, and identified two novel missense mutations, p.(Ala128Asp) (A128D) and p.(Phe240Leu) (F240L), that were predicted to be damaging to protein function. In the present work we aimed to investigate the potential oncogenic role of these mutations, comparing to that of the recurrent G84E mutation and wild-type *HOXB13*. We induced site-directed mutagenesis in a *HOXB13* expression vector and established *in vitro* cell models of prostate carcinogenesis with stable overexpression of either the wild-type or the mutated *HOXB13* variants. By performing *in vitro* assays we observed that, while the wild-type promotes proliferation, also observed with the F240L variant along with a decrease in apoptosis, the A128D mutation decreases apoptosis and promotes anchorage independent growth. No phenotypic impact was observed for the G84E mutation in the cell line model used. Our data show that specific *HOXB13* mutations are involved in the acquisition of different cancer-associated capabilities and further support an oncogenic role for *HOXB13* in prostate carcinogenesis.

## INTRODUCTION

A hereditary component is estimated to be present in 5-10% of all prostate cancers [1,2]. Although some cancer predisposition syndromes are known to increase the risk of prostate cancer development, namely the hereditary breast/ovarian cancer and Lynch syndromes [3], most cases of suspected hereditary prostate cancer remain without a molecular diagnosis. Recently, *HOXB13* was identified as a site-specific susceptibility gene for prostate cancer when Ewing and his colleagues found a recurrent germline mutation (G84E, rs138213197) in men of European descent, which co-segregated with the disease in affected families [4]. This association between the *HOXB13* G84E variant and an increased prostate cancer risk has been confirmed by other groups [5–13]. Although this variant only accounts for a small fraction of the prostate cancer cases, it confers an increased relative risk of 4.51-fold and is overrepresented in early-onset and familial prostate cancer [4,14,15]. Other *HOXB13* variants have also been found in different ethnic groups, suggesting allelic heterogeneity in different populations [4,16,17].

The *HOX* genes codify a family of transcription factors that are very important regulators of the positional identity of the organs and tissues in the anterior-posterior axis during embryonic development [18–21]. They are known to regulate cell proliferation and differentiation during embryogenesis and organogenesis, showing spatial and temporal colinearity [22–24]. Some *HOX* genes continue to be expressed in adult tissues that maintain developmental plasticity [19,21], and their deregulation has been associated with many types of cancers, such as those of the prostate, breast, ovary, endometrium, lung, kidney, colorectum, and pancreas [21,22,25–28]. *HOX13* paralogs are particularly important for the prostate gland development; however, *HOXB13* is the only that maintains a high expression level in adults, being confined to the rectum, distal colon and prostate, in an androgen-independent fashion [4,23,29–31]. Despite its relevance for the development of the normal prostate [23,32,33], its exact function is not yet fully understood, nor its role in prostate carcinogenesis. In fact, the role of *HOXB13* in prostate cancer development remains highly controversial, since it has been suggested to act both as an oncogene and as a tumor suppressor gene (TSG), and its role seems to be dependent on the cell type and on the cellular environment (androgen stimulation and androgen receptor (AR) status) [4,18,20,31,34]. Moreover, the biological impact of the reported *HOXB13* mutations has not yet been described.

With the intent to address the prevalence of *HOXB13* mutations among prostate cancer patients of the Portuguese population, we have recently sequenced the entire *HOXB13* coding region in 462 patients with early-onset and/or familial/hereditary prostate cancer and found two novel missense mutations – c.383C>A, p.(Ala128Asp) (A128D) and c.720C>A, p.(Phe240Leu) (F240L) – predicted to affect protein function by *in silico* analysis [17]. Nevertheless, the biological consequences and the mechanisms by which these novel variants promote carcinogenesis, as well as those of the recurrent G84E variant, still need to be explored. In the present study, we evaluated the oncogenic role of these *HOXB13* mutations using *in vitro* cell models of prostate carcinogenesis.

## RESULTS

### Establishment of the *in vitro* models to study the role of *HOXB13* mutations in prostate carcinogenesis using the PNT2 cell line

**Figure 1:**
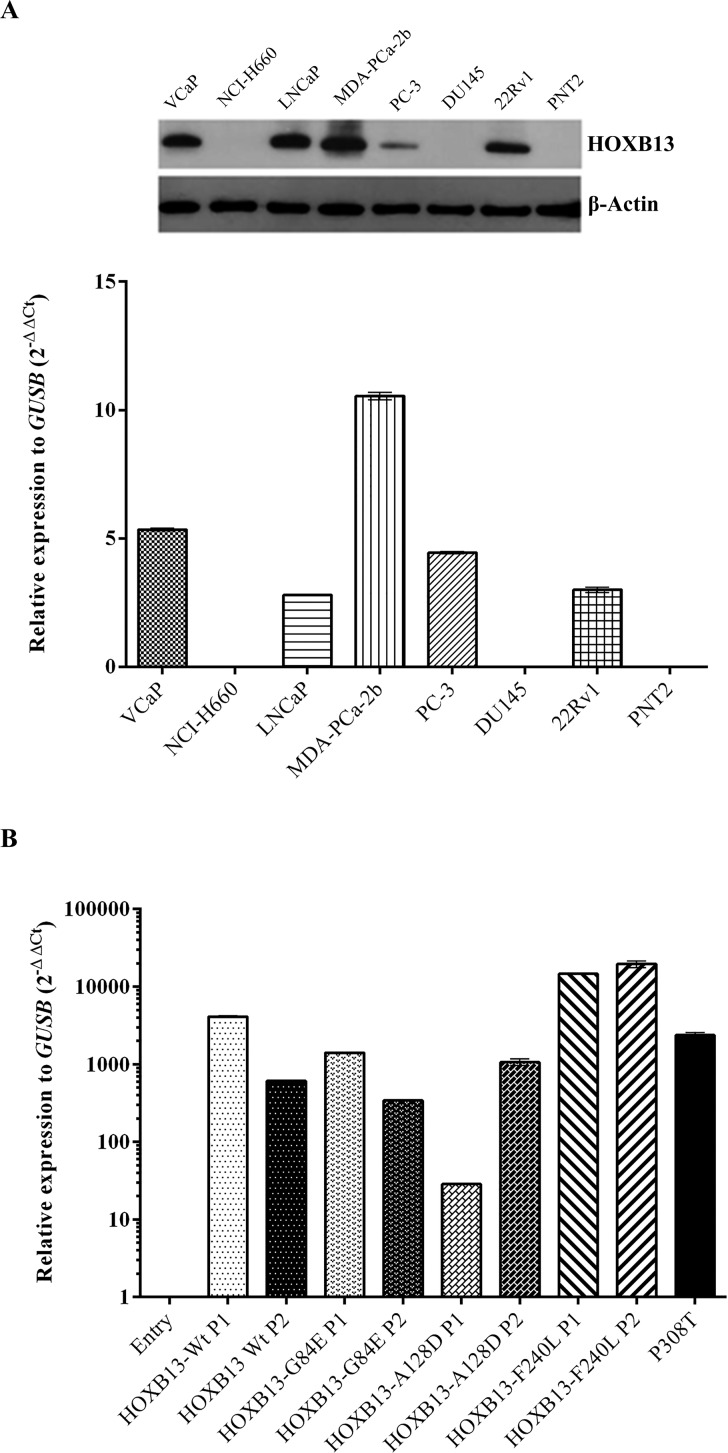
HOXB13 expression in cell lines and PNT2-derived cell line models (**A**) HOXB13 expression in eight prostate cell lines evaluated by immunoblotting and qRT-PCR. (**B**) HOXB13 mRNA expression after stable overexpression in PNT2 cells and comparison with the prostate carcinoma from one HOXB13 A128D mutation carrier (P308T). A negative control (Entry) and two polyclonal cell populations (P1 and P2) were chosen for each HOXB13 expressing vector.

To choose the most appropriate prostate cell line model to induce *de novo* expression of *HOXB13* and its mutated forms, *HOXB13* expression was evaluated by qRT-PCR and immunoblotting in eight prostate cell lines (Figure 1A). In the NCI-H660, DU145 and PNT2 cell lines no *HOXB13* was detected, in opposition to the other prostate cancer cell lines, with MDA-PCa-2b being the one with the highest expression levels. Considering the non-malignant characteristics of PNT2 cells [35] and the absence of *HOXB13* mutations in this cell line model (as confirmed by Sanger sequencing; data not shown), this cell context was selected to evaluate the oncogenic properties of *HOXB13* mutations. After transfection of PNT2 cells with the four different expression vectors (HOXB13-Wt, HOXB13-G84E, HOXB13-A128D and HOXB13-F240L; S1 Figure), overexpression of *HOXB13* in the transformed cell populations was confirmed by qRT-PCR. For each vector, the two populations with higher *HOXB13* expression were selected to proceed with functional studies. The expression levels of the established cell models were similar to those found in normal prostate tissues and tumor samples, including the tumor P308T from a *HOXB13* A128D mutation carrier (Figure 1B; S2 Figure). Regarding the three Entry (control) populations that were established, basal and equivalent *HOXB13* expression levels were detected (not shown), which lead us to select randomly one population for further studies.

### The *HOXB13* A128D and F240L mutations induce phenotypic alterations associated with early-stages of carcinogenesis

**Figure 2:**
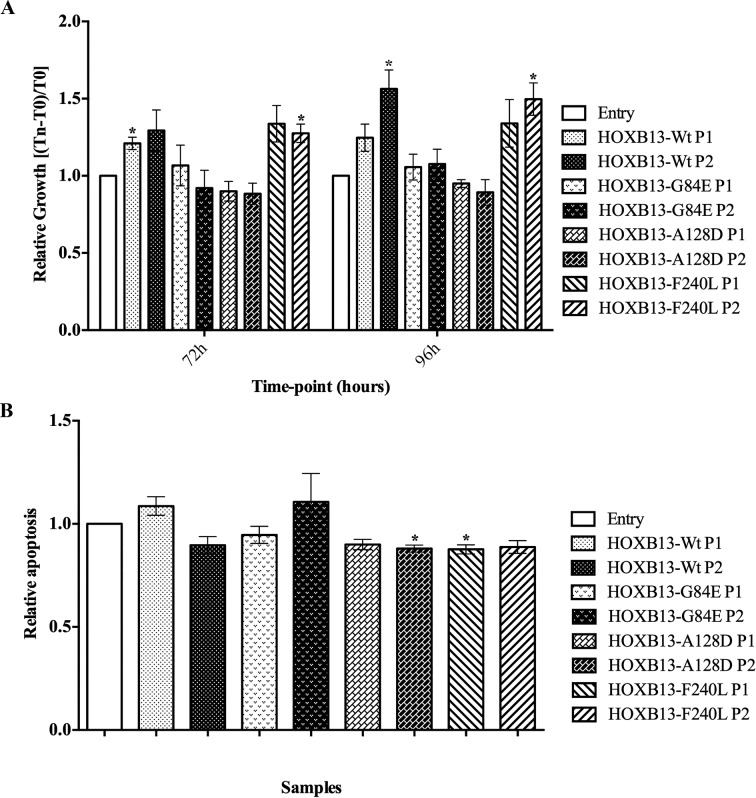
Impact of de novo overexpression of HOXB13-Wt and HOXB13 variants in proliferation and apoptosis (**A**) Quantification of cell viability by the MTT assay in PNT2-derived cells, at two time-points. (**B**) Quantification of apoptotic rates in PNT2-derived cells, evaluated after 96h in culture. Bars represent standard deviation. Results are shown for each HOXB13-expressing population relative to the control (Entry), from three independent experiments. Statistically significant *p* values (<0.05) are showed by an asterisk.

To evaluate the impact of *HOXB13* mutations in characteristics of tumor cells associated with early-stages of carcinogenesis, both the proliferation and the apoptosis levels were analyzed using the MTT and APOPercentage assays, respectively (Figure 2). The overexpression of the wild-type form of *HOXB13* was shown to have a growth stimulatory effect on PNT2 cells, with no changes in the apoptosis levels. In the MTT assay, despite the tendency observed at both time-points, this effect only reached statistical significance at 72h for one of the overexpressing populations (P1; *p*=0.035) and at 96h for the other (P2; *p*=0.044). Contrarily, overexpression of the A128D mutated form led to a decrease in the apoptosis rate, with statistical significance for P2 population (*p*=0.02) and borderline significance for P1 (*p*=0.06), but it did not affect proliferation. On the other hand, the F240L variant showed an impact in both cell proliferation and apoptosis, reaching statistical significance for P2 in proliferation (72h, *p*=0.044; 96h, *p*=0.026) and for P1 in apoptosis (*p*=0.03). The G84E mutation did not seem to alter either proliferation or apoptosis.

### The *HOXB13* A128D mutation promotes the acquisition of cellular properties involved in tumor progression

**Figure 3:**
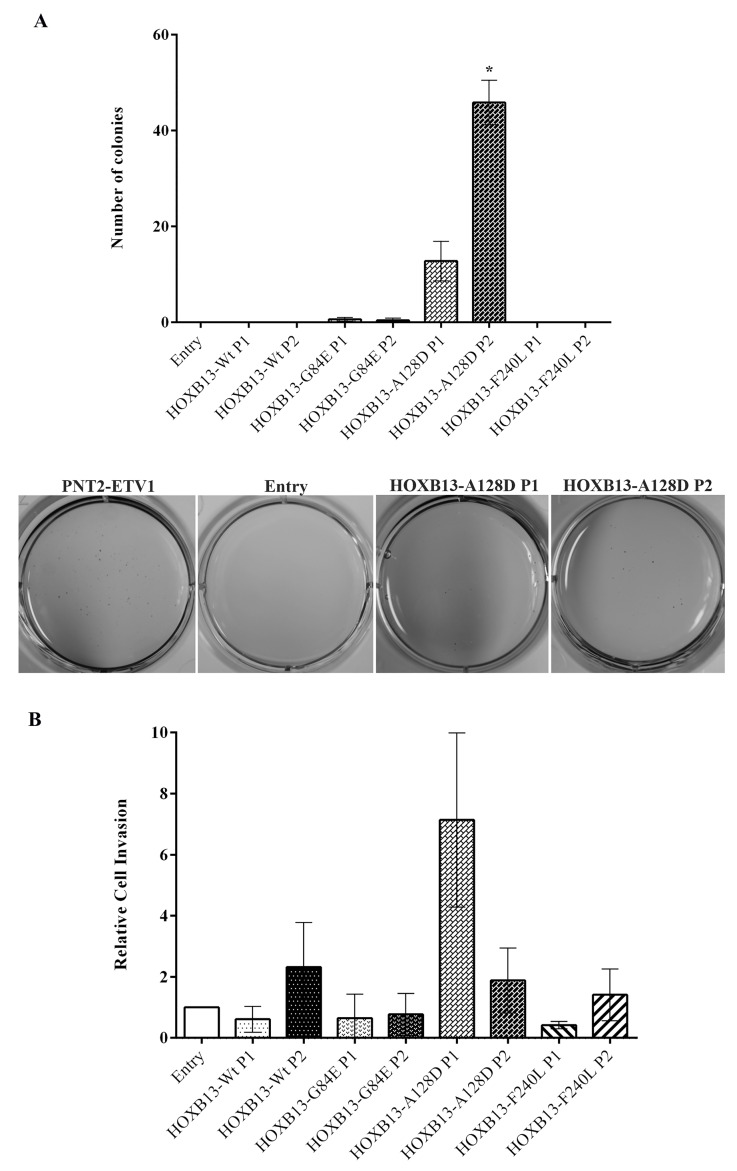
Impact of de novo overexpression of HOXB13-Wt and HOXB13 variants in anchorage-independent growth and invasion (**A**) Quantitative analysis (top) and qualitative visualization (bottom) of anchorage-independent growth by the soft agar colony formation assay, evaluated after 21 days in culture. (**B**) Quantitative analysis of cell invasion using Matrigel Invasion Chambers, after 48h in culture. Bars represent standard deviation. Results are shown for each HOXB13-expressing population relative to the control (Entry), from three independent experiments. Statistically significant p values (<0.05) are showed by an asterisk.

The oncogenic potential of *HOXB13* mutations in phenotypic changes associated with tumor progression was explored by evaluating anchorage independent growth and invasion *in vitro* (Figure 3). Only the mutated form A128D promoted cells' ability to grow without attachment, with both populations forming colonies that were visible to the naked eye (although results were only significant for P2; *p*=0.01). Regarding invasion, we observed large deviations between experiments. Nevertheless, one of the cell populations harboring the A128D variant (P1) showed a tendency for increased cell invasion, but the effect did not reach statistical significance.

## DISCUSSION

*HOXB13*, a member of the homeobox superfamily of transcription factors, is very important for normal prostate development and is the only *HOX13* paralog that continues to be expressed through adulthood [23,29,30,32]. Nevertheless, there are still uncertainties concerning its pattern of expression during prostate cancer progression. In this study we did not observe significant differences in *HOXB13* expression between prostate tumors and NPTs, neither among prostate tumors from different molecular subtypes defined by ETS fusion genes (S3 Figure). Although we have not found other reports that have compared the *HOXB13* expression in different ETS subtypes, our results are in agreement with other studies comparing carcinomas with non-tumor samples [18,20,34]. Additionally, those studies reported that different populations of HOXB13-negative and HOXB13-positive cells could be observed in the same tumor. On the other hand, other studies reported a higher expression of *HOXB13* in tumors when compared to the normal prostate [36–39]. Despite this controversy, it was demonstrated that hormone-refractory tumors overexpress *HOXB13* when compared to androgen-dependent carcinomas and its overexpression has been shown to promote prostate cancer invasion and metastasis [20,40]. Furthermore, *HOXB13* expression was associated with a more aggressive prostate cancer phenotype and was considered an independent predictor of unfavorable outcome [39].

With the recent description of *HOXB13* germline mutations predisposing to prostate cancer development [4,16,17], the relevance of *HOXB13* for prostate carcinogenesis gained a new perspective. Although found in only up to 5% of the prostate cancer cases of European descent showing familial clustering, the G84E mutation was the first genetic variant independently validated to be associated with site-specific hereditary prostate cancer predisposition [4–6,12,13]. In a recent work, we screened the *HOXB13* gene for germline mutations in 462 Portuguese patients with early-onset and/or familial/hereditary prostate cancer and found two novel mutations, A128D and F240L, which seem to confer an increased risk for prostate cancer development [17]. However, the biological mechanisms by which these variants promote carcinogenesis have not been explored. Given the importance of *HOXB13* in prostate biology and the debate surrounding its behavior as a TSG or as an oncogene, we aimed to gain insight into the biological consequences of the new A128D and F240L variants comparing with the G84E mutation and the wild-type *HOXB13*.

In order to select our study cell model, we evaluated *HOXB13* expression both at the mRNA and protein level in eight prostatic cell lines. The pattern of expression of *HOXB13* in our cell lines was similar to that described in the literature, reflecting the positive association between the expression of AR and *HOXB13* [31]. In fact, AR-positive cell lines (VCaP, LNCaP, MDA-PCa-2b and 22Rv1) [20,31] show high *HOXB13* expression (both at mRNA and protein), while AR-negative (NCI-H660, PC-3, DU145 and PNT2) [31,41,42] show low to absent expression. Considering the non-tumorigenic nature of the PNT2 cells and the absent expression of *HOXB13*, we selected these cells to induce *de novo* overexpression of *HOXB13* wild-type and its mutated forms G84E, A128D and F240L. Using our established cell models, we observed that *de novo* overexpression of wild-type *HOXB13* in this AR-negative cell line increases its proliferative phenotype. This observation is consistent with another study using the LNCaP cell line, where *de novo* overexpression of *HOXB13* markedly promoted cell proliferation in the absence of androgen stimulation [20]. According to Kim and collaborators, these results could explain why some patients relapse after androgen ablation, since *HOXB13* expression seems to be maintained in those tumors [20,34].

Regarding the oncogenic potential of specific *HOXB13* mutations, we here describe their involvement in different cancer-associated capabilities. The A128D variant was shown to be involved in the acquisition of characteristics associated with both early-stage and advanced prostate cancer cells, by decreasing apoptosis and promoting anchorage independent growth. On the other hand, the F240L variant seems to induce phenotypic changes involved in early carcinogenesis, promoting proliferation and decreasing apoptosis. Although in general we have not observed a dramatic phenotypic impact of *HOXB13* mutants (except for A128D in anchorage independent growth), the expression levels induced in our cell models are equivalent to those found in the tumors of our prostate cancer series (including the tumor from a *HOXB13* A128D mutation carrier), which lead us to infer that the established *in vitro* models would reproduce the HOXB13-dependent tumor biology. Although *in silico* analyses showed equivalent pathogenicity scores between G84E and the two novel variants here studied [17], we did not find differences between the established populations with the *HOXB13* G84E mutation and the control cells in any of the functional assays performed. Nevertheless, as we have only used one cell line model and no androgen stimuli was performed, the mechanism by which the *HOXB13* G84E mutation promotes prostate carcinogenesis remains unclear. In fact, Kim *et al*. reported an opposite impact of *HOXB13* in the proliferative potential of LNCaP cells with the presence or absence of androgen stimuli [34]. Curiously, the LNCaP cell line harbors a *HOXB13* mutation, L144P, which is located in one of the MEIS interacting domains, a functional domain equivalent to that potentially affected by the G84E mutation [4].

We conclude that the *HOXB13* A128D and F240L variants identified in Portuguese patients with early-onset/hereditary prostate cancer show oncogenic mechanisms more consistent with gain of function mutations. Although the evidence is less clear for the G84E variant, the phenotypic impact of *HOXB13* overexpression, the partially different oncogenic properties of the different mutations, the lack of truncating mutations, and the absence of loss of heterozygosity whenever tumors were tested [4,17], all concur with an oncogenic role of *HOXB13* in prostate carcinogenesis.

## MATERIALS AND METHODS

### Cell lines

The cell lines used in this study were VCaP, NCI-H660, LNCaP, MDA-PCa-2b, PC3, DU145, 22Rv1 and PNT2. DU145 cells were acquired from the German Resource Centre for Biological Material (DSMZ, Braunschweig, Germany) and grown at regular growth conditions in RPMI-1640 medium (GIBCO®, Invitrogen, Carlsbad, CA, USA), supplemented with 10% fetal bovine serum (FBS, GIBCO®) and 1% penicillin/streptomycin (GIBCO®). The origin and growth conditions of the remaining cell lines were previously described [43,44]. Conventional G-banding karyotyping was previously performed to confirm cell identity [44]. All prostate cell lines were routinely tested for *Mycoplasma spp.* contamination using the PCR Mycoplasma Detection Set (Clontech Laboratories Inc.).

### Selection of the study cell model

To explore the oncogenic potential of *HOXB13* and its mutated forms, we established cell models of prostate tumors by inducing de novo overexpression of the wild-type (Wt) *HOXB13* and the G84E, A128D and F240L mutated forms. To select a cell line with low or basal *HOXB13* expression levels, we first evaluated *HOXB13* expression, both at RNA and protein level, in eight prostate cell lines available at our laboratory (VCaP, NCI-H660, LNCaP, MDA-PCa-2b, PC3, DU145, 22Rv1 and PNT2), using quantitative real-time reverse transcription PCR (qRT-PCR) and immunoblotting, respectively. As a second selection criterion, and since we sought to study the effect of specific *HOXB13* mutations in prostate carcinogenesis, the chosen cell line model could not harbor any other mutation in this gene. Therefore, in the cell lines for which there was no available data in the literature (namely for NCI-H660 and PNT2 cells), sequencing of the entire *HOXB13* coding region was performed.

### Quantitative real-time reverse transcription PCR (qRT-PCR)

To evaluate *HOXB13* relative expression levels in the eight wild-type prostatic cell lines and in the newly established cell populations, RNA was extracted from subconfluent cells using the TRIzol® Reagent (Ambion® by Life Technologies, Foster City, CA, USA) and converted into cDNA using the RevertAid H Minus First Strand cDNA Synthesis Kit (Thermo Fisher Scientific, Rockford, IL, USA), according to the manufacturer´s instructions. For the analysis of *HOXB13* mRNA expression levels in tumor and normal prostate tissue (NPT) samples, cDNA was synthetized using the TransPlex Whole Transcriptome Amplification kit (Sigma-Aldrich, St Louis, MO, USA), as previously described [45]. *HOXB13* primers and probe (primer forward: 5´-GTTGCCAGGGAGAACAGAAC-3´; primer reverse: 5´-TGTACGGAATGCGTTTCTTG-3´; Taqman probe: 5´Fam-AAGGCAGCATTTGCAGACTCCAGC-3´Tamra) were designed using the Primer3 software (v.0.4.0) [46] and acquired from Metabion (Martinsried, Germany). The reactions were performed in triplicate and the beta-glucuronidase (*GUSB*) housekeeping gene was used as an endogenous control, with probes acquired as pre-developed assays from Applied Biosystems (by LifeTechnologies). *HOXB13* relative expression levels in the wild-type prostatic cell lines were quantified using the comparative threshold cycle method (ΔCt) [47] and the ΔΔCt method was used for the newly established cell populations [48], by calibrating *GUSB* normalized *HOXB13* expression values from each cell population to the expression levels of the control population.

### Western blotting

Protein extracts were obtained from subconfluent cells using RIPA lysis buffer with protease inhibitors (Santa Cruz Biotechnology, Heidelberg, Germany) and concentrations were measured by the Pierce BCA protein assay (Thermo Fisher Scientific). Immunoblotting was carried out under standard procedures and specific detection of HOXB13 was achieved by incubation with a mouse anti-HOXB13 monoclonal antibody (1:1000, mAbcam53931, Abcam, Boston, MA, USA). To control protein loading, an anti-β-actin antibody (1:8000, clone AC-15, A1978, Sigma-Aldrich) was used. Signals were amplified and detected as previously described [44].

### Sanger sequencing

DNA was extracted from NCI-H660 and PNT2 cells using the illustra TriplePrep kit (GE Healthcare, Life Sciences, Cleveland, OH, USA) according to the manufacturer´s instructions, and *HOXB13* sequencing was performed as previously described [17].

### Site-directed mutagenesis and construction of *HOXB13* mutants

A *HOXB13* expression vector (RC209991, Origene, Rockville, MD, USA) was expanded in Stellar™ Competent Cells (Clontech Laboratories, Inc., Saint-Germain-en-Laye, France) and used as a template to create the *HOXB13* mutants using the QuikChange II Site-Directed Mutagenesis Kit (Agilent Technologies, Santa Clara, CA, USA), according to the instructions. Briefly, to induce the G84E, A128D and F240L mutations, three sets of mutagenic primers (Sigma-Aldrich) were designed (S1 Table) using the Quick-Change Primer Design Program (Agilent Technologies). The first step of the site-directed mutagenesis consisted on mutant strand synthesis by PCR using the mutagenic primers, according to the manufacturer´s recommendations. Besides the three mutant strand synthesis reactions, an additional reaction was done with the control vector pWhitescript 4.5-kb to evaluate mutagenesis efficiency. Amplified products were then treated with *DpnI* at 37°C for one hour, which digests the parental methylated and hemimethylated DNA template, thus allowing the selection of the newly synthetized mutated DNA. Finally, XL1-Blue supercompetent cells (Agilent Technologies) were transformed with the vectors containing the desired mutations and with a control plasmid, pUC18, to evaluate the efficiency of the transformation.

An empty vector (Entry) was also created to serve as a control. For that purpose, the open reading frame (ORF) was excised from the original *HOXB13* vector with FastDigest Sal I and FastDigest *Xho I* (both from Thermo Fisher Scientific) at 37°C for one hour, and the plasmid religated with T4 DNA Ligase (Thermo Fisher Scientific) at 22°C for one hour. Stellar™ Competent Cells were then transformed and expanded.

After transformation, DNA from each plasmid was extracted using the NucleoSpin® Plasmid kit (Macherey-Nagel, Düren, Germany) following the manufacturer's instructions, and their identity was confirmed by Sanger sequencing using the primers supplied with the vector (S2 Table). To simplify nomenclature, the obtained vectors will be referred as Entry (for the empty vector), HOXB13-Wt (for the original wild-type *HOXB13* vector), and HOXB13-G84E, HOXB13-A128D and HOXB13-F240L (for the *HOXB13* mutated vectors).

### Stable *de novo* overexpression of *HOXB13*-Wt and *HOXB13* mutants in PNT2 cells

PNT2 cells were transfected with the four *HOXB13* expression vectors (Wt and mutated) and with the control vector (Entry) using TurboFectin 8.0™ transfection reagent (Origene). Briefly, one day before transfection, PNT2 cells were plated in 60mm dishes at a density of 1.5×105 in complete growth medium, in order to reach a confluence of approximately 60% at the day of transfection. For each transformation, 250μL of Opti-MEM medium (GIBCO®) were mixed with TurboFectin 8.0™ and incubated at room temperature for five minutes. The vector DNA was added and incubated at room temperature for 30 minutes. In order to achieve the best transfection efficiency, three different ratios of TurboFectin:DNA were used for each vector: 3:1 (7.5μL of TurboFectin for 2.5μg of DNA), 3:2 (7.5μL of TurboFectin for 5μg of DNA) and 6:1 (15μL of TurboFectin for 2.5μg of DNA). The TurboFectin:DNA containing mixtures were then added dropwise to the cells and incubated for 48 hours at regular growth conditions. At this point, cells were exposed to selective pressure by adding regular growth medium supplemented with 450µg/µL of G418 (GIBCO®). Four to five weeks later stable cell populations were obtained.

### Cell proliferation assay

Cell proliferation was evaluated with the MTT colorimetric assay. PNT2-derived populations were seeded in 96-well plates at a density of 1×104 cells per well and incubated at regular growth conditions for 72 and 96 hours. At each time point, cells were exposed to the MTT solution (Sigma-Aldrich) for two hours. Formazan crystals were dissolved with DMSO (Merck, Darmstadt, Germany) and the absorbance was measured in a microplate reader (Fluostar Omega, BMG Labtech, Ortenberg, Germany), as previously described [44]. Cell growth was calculated using the formula (Tn-T0)/T0 [44] and relative growth was estimated by normalizing the values of each *HOXB13*-expressing population to the control (Entry).

### Apoptosis assay

PNT2-derived cell populations were seeded in 96-well plates at a density of 1×104 cells per well and incubated at regular growth conditions for 96 hours. The APOPercentage kit (Biocolor, Carrickfergus, UK) was used to evaluate apoptosis, following the manufacturer's instructions. Absorbance was measured in a microplate reader and an average value for each cell population was obtained from nine replicate wells, as previously described [44]. Relative apoptosis was obtained by normalizing the values of each *HOXB13*-expressing population to the control (Entry).

### Soft agar colony formation assay

To evaluate anchorage independent growth, the soft agar colony formation assay was used. A bottom layer of 1.2% low-melting agarose (Lonza by VWR, Radnor, PA, USA) in normal culture medium was prepared in six-well culture plates, and, on top, a layer of 0.2% agarose containing 1×104 cells was plated and covered with the same culture medium. Cells were incubated at regular growth conditions for three weeks. At this time-point, cells were stained with 0.05% crystal violet and the number of colonies was counted by direct visualization. PNT2-ETV1 cells were used as a positive control, as previously described [44].

### Invasion assay

The invasive potential of PNT2-derived cells was evaluated *in vitro* using the BD BioCoat™ Matrigel™ Invasion Chambers (BD Biosciences, Billerica, MA, USA), according to the manufacturer´s instructions. Complete growth medium supplemented with 20% FBS was added to the lower chambers and on top, in the upper chambers, cell suspensions in complete growth medium (5% FBS) were seeded (2×104 cells per well) and incubated for 48 hours at regular growth conditions. At this time point, non-invading cells were swabbed from the upper surface of the chambers whereas invading cells, on the lower side, were stained and visualized in a fluorescence microscope. Invaded cells were counted in representative microscope fields [44] and relative cell invasion was obtained by normalizing values of each *HOXB13*-expressing population to the control (Entry). The invasive TPC-1 cells were used as a positive control.

### Statistical analysis

For all *in vitro* assays, three independent experiments were performed, each including triplicate wells per condition (unless otherwise stated). The significance of the data was evaluated with the Student's *t* test using GraphPad Prism 6 and results were considered statistically different when *p* values were below 0.05.

## SUPPLEMENTARY MATERIALS FIGURES AND TABLES


