# Supplementary material for: Oncogenic mechanisms of HOXB13 missense mutations in prostate carcinogenesis
Source: Oncoscience. 2016 Oct 31;3(9-10):288–96. doi: 10.18632/oncoscience.322 (PMC5116946; doi:10.18632/oncoscience.322)
Supplement: Supplementary file 1 [file oncoscience-03-288-s001.pdf]

SUPPLEMENTARY MATERIALS

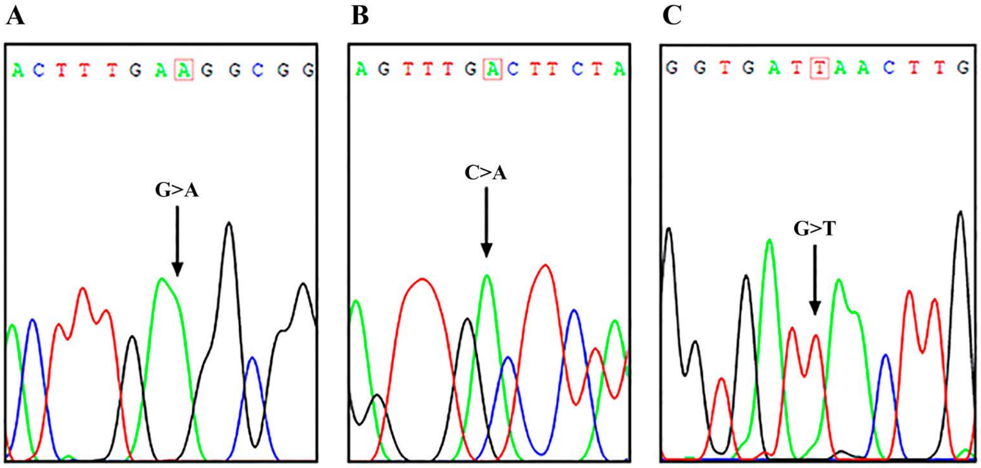

S1 Figure – Sanger sequencing validation of the induced site-directed mutagenesis. DNA sequence chromatograms corresponding to the forward strand of the vectors G84E (A) and A128D (B) and to the reverse strand of the vector F240L (C).

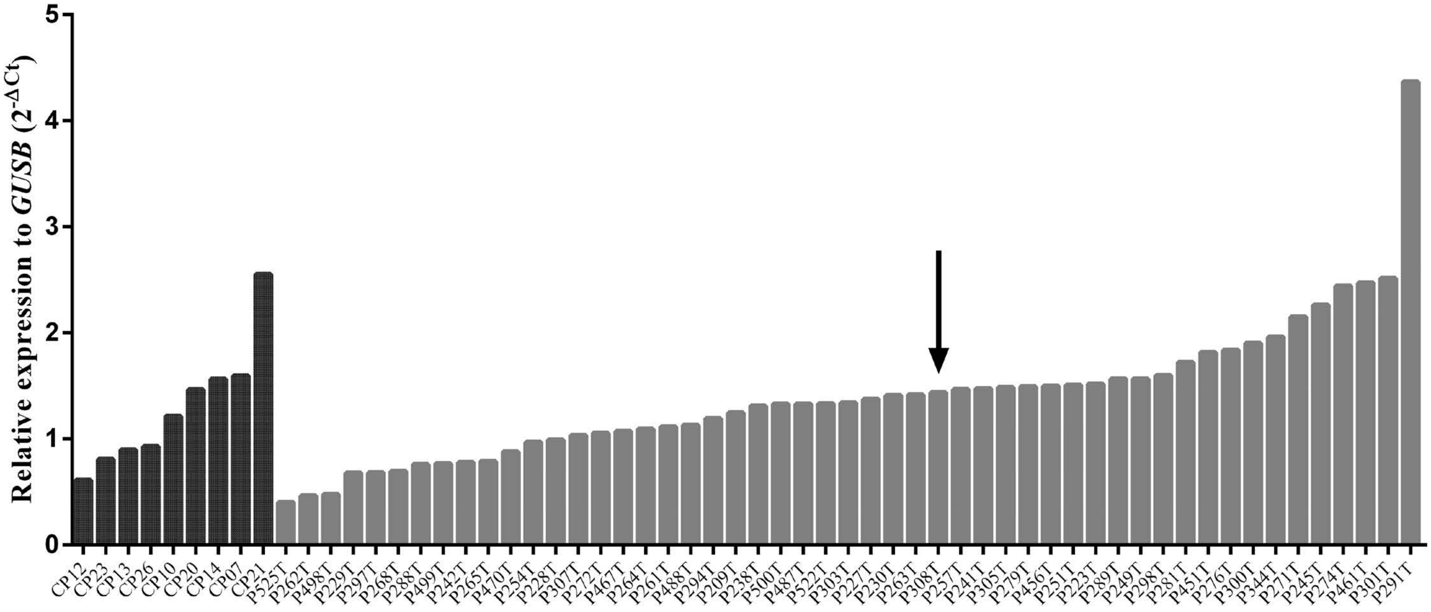

S2 Figure – HOXB13 mRNA expression evaluated by qRT-PCR in nine NPTs and in 51 prostate tumor samples. The 51 tumor samples were selected from a consecutive series of 200 clinically localized prostate cancer to represent the various ETS molecular subtypes [49] and the nine NPTs have been previously obtained from cystoprostatectomy specimens of bladder cancer patients [43]. The tumor from the carrier of the HOXB13 A128D mutation (P308T) is pointed with an arrow.

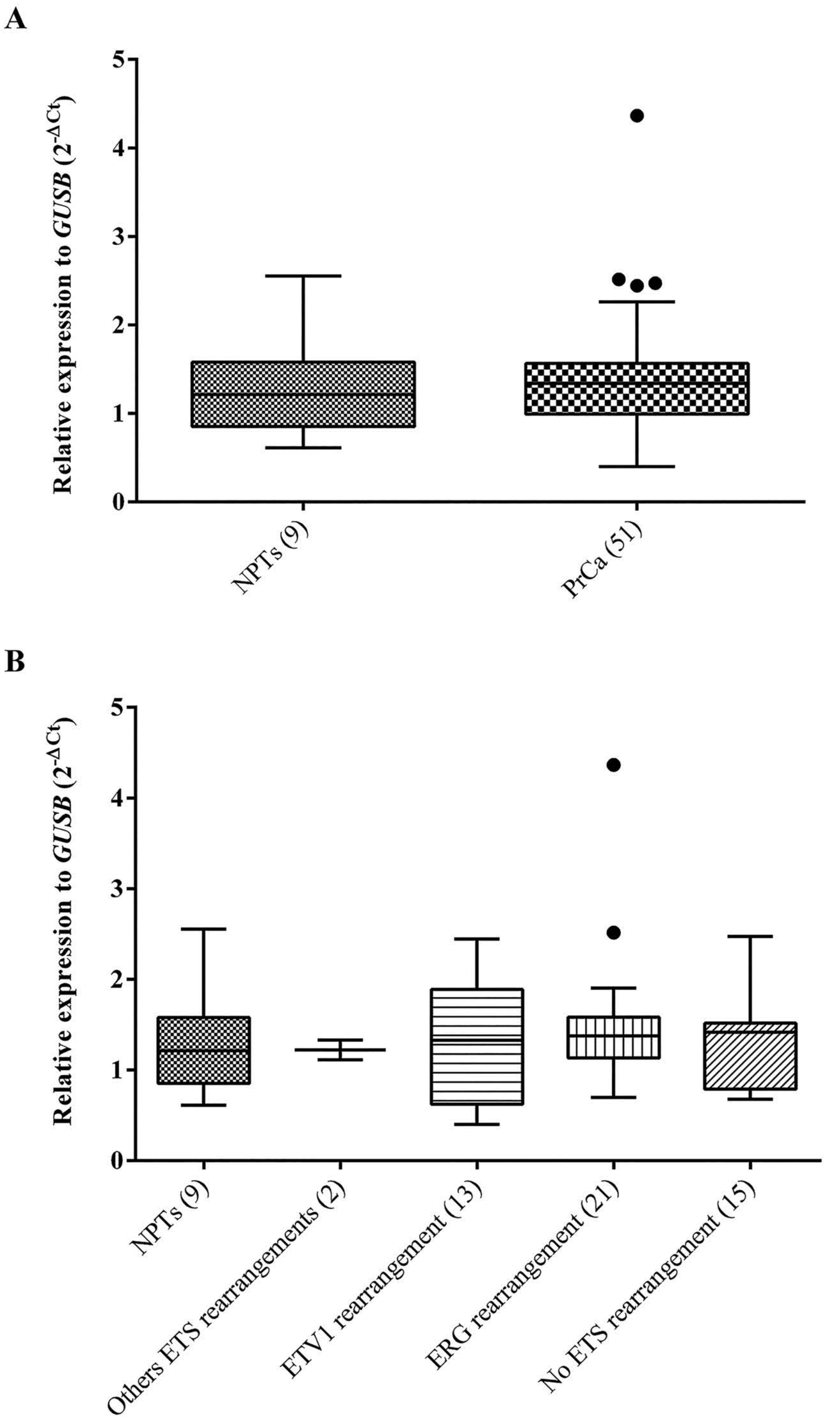

S3 Figure – HOXB13 mRNA expression evaluated by qRT-PCR. (A) HOXB13 expression levels in NPT samples vs prostate carcinomas (Mann-Whitney test; $p>0.05$). (B) HOXB13 expression levels in different prostate cancer ETS subgroups (Kruskal-Wallis test; $p>0.05$).

S1Table–Sequences of the primers used to induce site-directed mutagenesis.

Primers	Sequence 5' to 3'
G84E-F	GGTTACTTTGAAGGCGGGTACTACTCCTGCC
G84E-R	GGCAGGAGTAGTACCCGCCTTCAAAGTAACC
A128D-F	CGCCCCACTGAGTTTGACTTCTATCCGGG
A128D-R	CCCGGATAGAAGTCAAACTCAGTGGGGCG
F240L-F	GCGGCTAACAAGTTAATCACCAAGGACAAGAGGC
F240L-R	GCCTCTTGTCCTTGGTGATTAACTTGTTAGCCGC

S2Table–Sequences of the primers used for Sanger sequencing of the plasmids.

Primers	Sequence 5' to3'
VP1.5(Forward)	GGACTTTCCAAAATGTCG
XL39(Reverse)	ATTAGGACAAGGCTGGTGGG
